# Persistent Vulnerabilities in Neurons Following Lead (Pb) Exposure and Implications for Alzheimer's Disease

**DOI:** 10.1002/alz70855_107301

**Published:** 2025-12-25

**Authors:** Junkai Xie, Shichen Wu, Han Zhao, Jennifer L Freeman, Chongli Yuan

**Affiliations:** ^1^ Purdue University, West Lafayette, IN, USA

## Abstract

**Background:**

Exposure to environmental neurotoxicants such as lead (Pb) during critical developmental periods and adulthood has been linked to persistent neurological deficits and pathological features resembling Alzheimer's disease (AD). Epidemiological studies have reported associations between Pb exposure and an increased risk of AD later in life, a link further supported by animal models. However, the molecular mechanisms underlying this association remain poorly understood.

**Method:**

In this study, we utilized hiPSC‐derived cortical neurons to investigate the impact of Pb exposure at different life stages on the onset of AD‐like pathogenesis. We selected environmentally relevant Pb concentrations (0, 15, and 50 ppb) and assessed both immediate and persistent neurological effects using immunofluorescence, Western blotting, RNA sequencing, enzyme‐linked immunosorbent assay (ELISA), microelectrode array (MEA) and a “secondary hit” model.

**Result:**

Although neurite morphology remained largely intact, Pb‐exposed neurons displayed significant hyperactivity and mitochondrial dysfunction. Transcriptomic profiling identified differentially expressed genes enriched in oxidative phosphorylation and AD‐related pathways. Furthermore, Pb exposure led to an increase in phosphorylated Tau, Tau aggregates, and Aβ42/40 ratios—hallmarks of AD pathology. Notably, Pb‐exposed neurons exhibited increased susceptibility to AD‐relevant secondary stressors, including PHF‐Tau and the mitochondrial toxin MPP+, with these vulnerabilities persisting even after Pb withdrawal.

**Conclusion:**

These findings provide mechanistic insight into how Pb exposure may contribute to AD pathogenesis, highlighting potential molecular pathways that could mediate the increased AD risk observed in Pb‐exposed populations.

